# Test-Retest Reliability of Kinematic and Temporal Outcome Measures for Clinical Gait and Stair Walking Tests, Based on Wearable Inertial Sensors

**DOI:** 10.3390/s22031171

**Published:** 2022-02-03

**Authors:** Sofie Nilsson, Per Ertzgaard, Mikael Lundgren, Helena Grip

**Affiliations:** 1 Department of Rehabilitation Medicine and Department of Health, Medicine and Caring Sciences, Linkoping University, 581 83 Linköping, Sweden; sofie.nilsson@liu.se (S.N.); per.ertzgaard@liu.se (P.E.); 2 Department of Rehabilitation, Västervik Hospital, 593 33 Västervik, Sweden; mikael.lundgren@regionkalmar.se; 3 Department of Radiation Sciences, Biomedical Engineering, Umeå University, 901 87 Umeå, Sweden

**Keywords:** inertial sensor, healthy individuals, gait analysis, clinical practice

## Abstract

It is important to assess gait function in neurological disorders. A common outcome measure from clinical walking tests is average speed, which is reliable but does not capture important kinematical and temporal aspects of gait function. An extended gait analysis must be time efficient and reliable to be included in the clinical routine. The aim of this study was to add an inertial sensor system to a gait test battery and analyze the test-retest reliability of kinematic and temporal outcome measures. Measurements and analyses were performed in the hospital environment by physiotherapists using customized software. In total, 22 healthy persons performed comfortable gait, fast gait, and stair walking, with 12 inertial sensors attached to the feet, shank, thigh, pelvis, thorax, and arms. Each person participated in 2 test sessions, with about 3–6 days between the sessions. Kinematics were calculated based on a sensor fusion algorithm. Sagittal peak angles, sagittal range of motion, and stride frequency were derived. Intraclass-correlation coefficients were determined to analyze the test-retest reliability, which was good to excellent for comfortable and fast gait, with exceptions for hip, knee, and ankle peak angles during fast gait, which showed moderate reliability, and fast gait stride frequency, which showed poor reliability. In stair walking, all outcome measures except shoulder extension showed good to excellent reliability. Inertial sensors have the potential to improve the clinical evaluation of gait function in neurological patients, but this must be verified in patient groups.

## 1. Introduction

Gait analysis is an important part of the assessment of body function in neurological disorders. Stroke is one of the largest patient groups as one in four persons will suffer a stroke during their lifetime [1] and two-thirds of stroke survivors will have limited walking ability [2]. Improving the walking ability is one of the highest priorities in stroke rehabilitation, and early assessment and intensive training is needed for maximal recovery. Clinical tests assessing gait at a comfortable speed are used extensively to evaluate post-stroke patients, such as the timed 10-m walk test (10 mWT) and the 6-min walk test (6 mWT) [3]. A gait test focusing on fast gait is the timed 25-foot walk test (T25FW), but this is most commonly used in the assessment of multiple sclerosis [4]. Stair-walking tests, such as the 12-step ascend and descend test, provide important information about gait ability post-stroke under more demanding conditions [5]. The only outcome measure from all these gait tests is the average walking speed [3]. This outcome measure has high inter- and intra-rater reliability [6], but it does not capture the important aspects that explain the resulting gait velocity post-stroke, such as joint kinematics [7,8], stance phase time [9], temporal side asymmetries [10], arm swing during gait [11], or adaptions of gait patterns during fast gait [12].

The clinical gait assessment post-stroke could be improved by implementing wearable inertial sensors, i.e., miniaturized sensors, including three-dimensional accelerometers, gyroscopes, and/or magnetometers, in the clinical evaluation. A review concluded that the inclusion of wearable inertial sensors in the clinical 6 mWT provides a thorough and comprehensive assessment of gait function, without further burdening the patient [13]. It is most common to use a small number of inertial sensors, placed on the feet and/or shanks or the pelvis, to analyze temporal outcome measures [14,15] while full body models and kinematic variables are less commonly analyzed [14]. Still, kinematic measures from the lower body and arms are important in the assessment of stroke, as they give information about body function that may be used to grade stroke severity [11,16].

Despite the body of evidence on the reliability of inertial sensors [14,15,17], this technology is still seldom included in clinical examinations. The main reasons are that the protocols and outcome measures need to be standardized and validated for their specific area of use before they are implemented in clinical routines, especially since the reliability depends on the types of movement and segments that are analyzed, the sensor placement [18,19], and the computational choices for data extraction [20]. Further, the measurements and analyses must be time efficient, and selected outcome measures should give valid clinical information. The methodology for properly conducting and interpreting the exam is important, and a description of the reliability of the data obtained is required before its use in a clinical setting [21].

We hypothesize that inertial sensors could be added to a clinical test battery involving comfortable gait, fast gait, and stair walking, to extend the current outcome measure (average speed) with reliable kinematic and temporal information about gait performance, which is relevant for patients with neurological disorders, such as stroke. The aim of this study was therefore to analyze the test-retest reliability of outcome measures from inertial sensors placed on the lower body and arms, i.e., sagittal range of motion, sagittal peak joint angles, and stride frequency. As a first step, this was evaluated in a group of adult persons with normal walking ability.

## 2. Materials and Methods

### 2.1. Participants

In total, 22 persons with normal gait function participated in this study, including 12 females (mean 45 ± 12 years, BMI mean 22 ± 3) and 10 males (mean 38 ± 13 years, BMI mean 24 ± 2). All participants had right leg dominance according to the question “If you would shoot a ball on a target, which leg would you use?” [22]. The participants were recruited through convenience sampling by email to the staff where the study took place and to individuals related to members of the research team. The inclusion criterion was age from 18–70 years. The exclusion criterion was affected gait function because of injury, illness, or pain. This study was conducted according to the guidelines of the Declaration of Helsinki and approved by Regional Ethical Review Board in Umeå, Sweden (Dnr 2018/236-31, 7 August 2018). Written informed consent was obtained from each participant.

### 2.2. The Clinical Test Battery

This study was performed in two Swedish rehabilitation clinics, with two different physiotherapists (SN and ML), one at each clinic. The physiotherapists attended a joint training session prior to the study to ensure standardization of the verbal instructions given to participants, sensor placements, and measurement procedures.

Each participant was dressed in shorts, t-shirt, and socks with anti-slip. All participants completed 2 test sessions, with 3 to 6 days between each session. In each test session, three gait tests were performed with three trials each:

(1) Gait 15 m at a comfortable speed;
(2) Gait 15 m at a fast speed: the instruction was to “*walk as fast as possible without running or losing balance, as if you are in a hurry to catch the bus*”;
(3) Stair walking at a comfortable speed, stair ascending followed by stair descending repeated 3 times (step height 17.5 cm, step depth 27.3 cm). During stair walking, the participant held the dominant (right) hand on the handrail, since this is how it would be done in the clinic to minimize the risk of falls when assessing a patient with gait disorders.

**Figure 1:**
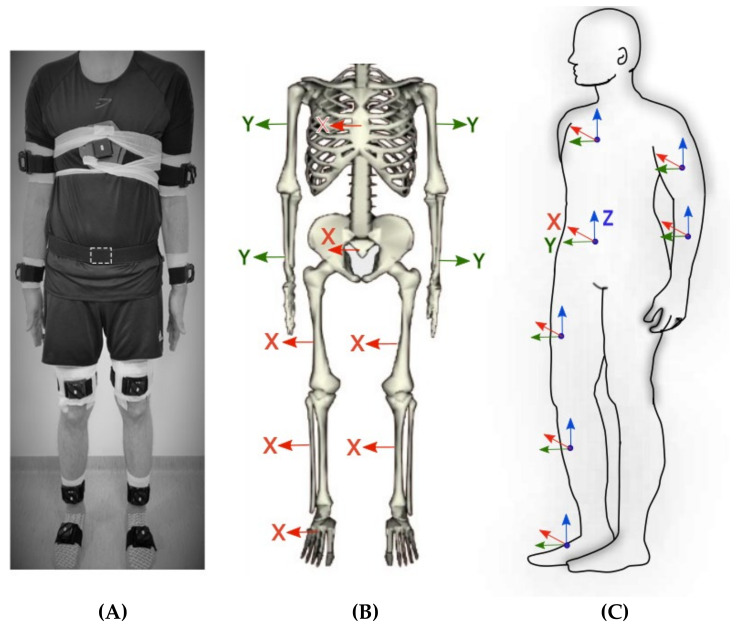
The (**left figure A**) illustrates the sensor placements on the thorax, upper and lower arms, legs, and feet. The person is standing in the neutral position. The pelvic sensor is placed on the back of the pelvis, and its position is marked with a dashed line. The orientation of the raw local *sensor* coordinate systems are shown in the segment model in the (**mid figure B**). The local *segment* coordinate system according to the segment model are illustrated in the (**right figure C**). For all segments, X pointed in the mediolateral direction (representing flexion-extension), Y pointed in the anterior direction (representing abduction-adduction), and Z in the inferior direction (representing inward/outward rotation).

Prior to each trial, the participant stood still in a standardized position, which was standing as straight as possible, with feet pointing forward a hip-width distance apart (as secured by a wooden block), looking forward, and with arms hanging straight along the side of the body with the thumbs pointed forward (Figure 1). The physiotherapist provided assistance to ensure that the standardized position was correct and gave verbal instructions to the participant if corrections were needed. The underlying assumptions are that in this standardized position, all body segments are aligned with gravity and have zero internal/external rotation.

### 2.3. Equipment and Sensor Placement

The portable inertial sensor-based system MoLab^TM^ (AnyMo AB, Umeå, Sweden) was used to register the movement with a sampling frequency of 128 Hz. The system’s accuracy and precision are about 2–3° for lower body movements in comparison to a 3D optical camera system (QTM, Qualisys AB) [23]. The system consisted of a battery-powered unit with a microprocessor that communicated with 12 sensors, each including an accelerometer, a magnetometer, and a gyroscope for 3D registration. The magnetometer data was excluded since the surroundings, as in many modern hospitals, contained magnetic material in the walls and floors, causing severe magnetic disturbances. The dynamic ranges of the 3D gyroscopes and accelerometers were ±300 degrees/s and ±10 g, respectively, with a resolution of 14 bits. The inertial motion sensor signals were wirelessly transmitted to a PC during the motion registration and were further analyzed with the MoLab^TM^ software.

The sensors were placed by the physiotherapist (S.N. or M.L.) on the participant in a standardized way using elastic straps and premade adapted holders. The sensors’ positions on the upper body were based on a previous study that analyzed optimized upper body sensor placements [18]. The sensor positions on the lower body were described in an earlier study [23] and were chosen based on experiences from gait measurements in an optical movement laboratory [24] to minimize soft tissue artefacts from muscle and skin motion, with the additional requirement that the anterior-posterior axis of each sensor should point in the forward direction during the standardized position. Wedges with different heights were used in the premade holders to ensure this. The reason for this requirement is further described in the data preprocessing section. The selected placements were (1) on the upper body over the manubrium, (2) on the upper arms at the distal part of the arm, at a distance from the elbow joint corresponding to one-third of the arm’s length, (3) on forearms dorsally at the distal end close to the ulnar process, (4) on the posterior pelvis at the mid-point between the right and left anterior superior iliac spine (ASIS), (5) on the right and left thigh three centimeters above the apex patella, (6) on the right and left shank below the m. gastrocnemius, and (7) on the dorsal surface of each foot over the midfoot (Figure 1).

### 2.4. Data Preprocessing

The segment model was a 3 degree-of-freedom model, defined so that each segment’s Z axis pointed in the participant’s inferior direction along the gravity vector, each X axis pointed in the medio-lateral direction (pointing toward the participant’s right side), and each Y axis pointed in the anterior direction when standing in the standardized position. This required a 90° rotation around Z on the sensor data from the arm sensors since these 4 sensors were placed so that Y pointed in the superior direction, and a 90° rotation around X on sensor data from the feet sensors, which were placed so that the sensor’s Y pointed in the vertical direction when standing in the neutral position.

The sensors’ three-dimensional orientation were then calculated using the Madwick fusion algorithm based on a quaternion representation [25]. The filter gain (controlling by which amount the accelerometer corrected the orientation estimated by the gyroscope) was set to 0.03. The first seconds of each trial, where the participant stood still in a standardized position, were used to initially remove gyroscopic drift and to align each sensor’s coordinate system in the frontal and sagittal planes, according to the segment model, and all rotations were set to zero. Direction cosine matrices (DCMs) were derived from the calculated quaternions. Joint angles were represented by relative DCMs, i.e.,

DCM_JOINT_ = DCM^T^_reference_segment_ · DCM_moving_segment_ (1)

Angular data was then computed from the resulting DCMs using the Cardan sequence XYZ, where X represents the movement in the sagittal plane, Y represents the movement in the frontal plane, and Z represents the movement in the transverse plane. Sagittal plane movement was selected for further analysis and included:

- Pelvis flexion-extension (computed from the pelvis sensor).
- Shoulder flexion-extension (computed from the upper arm sensor relative to the thorax sensor).
- Thorax flexion-extension (computed from the thorax sensor relative to the pelvis sensor).
- Hip flexion extension (computed from the thigh sensor relative to the pelvis sensor).
- Knee flexion-extension (computed from the shank sensor relative to the thigh sensor).
- Foot dorsiflexion-plantarflexion (computed from the foot sensor relative to the shank sensor). Note that foot dorsiflexion (foot moving upwards) and foot plantarflexion (foot moving downwards) are the clinical terms for the ankle joint movement.

**Figure 2:**
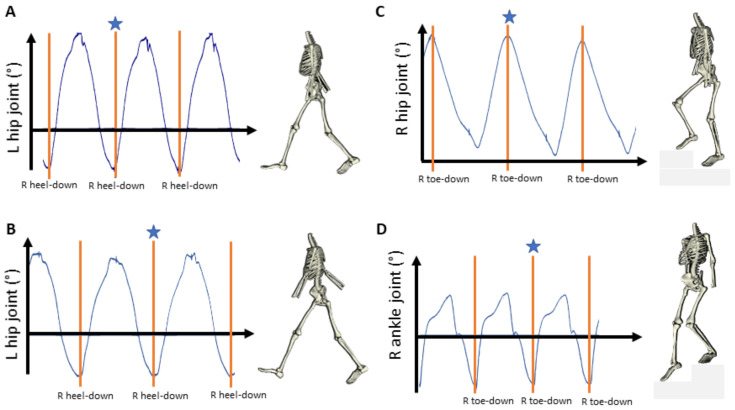
Example of the event settings for one of the study participants. Joint curves and skeletons are printed from the MoLab^TM^ analysis software. Image adjustments, such as adding vertical orange lines, gray shaded stairs, and axis legends, were done for graphical reasons only. The joint curve used for each event setting is shown for comfortable gait (**A**), fast gait (**B**), stair ascending (**C**), and stair descending (**D**), together with the animated segment model. The star marks the event at the time frame for which the segment model for the participant is displayed.

Kinematic and temporal variables were extracted for each trial. For level walking, the gait cycle, or stride, is defined as the phase between two consecutive heel strikes of the same limb (initial contact). The initial contact was computed by the event “heel-down” for fast and comfortable gait and was defined to occur when peak hip extension occurred in the contralateral side (Figure 2A,B). This definition was considered robust after comparisons with heel-down events from gait lab data (unpublished work).

During stair walking, the initial contact instead occurs when the toes are put down. The gait cycle for each side was hence defined to start and stop at “toe-down”. For stair ascending, toe-down was defined to occur at peak hip flexion on the ipsilateral side (Figure 2C). For stair descending, toe-down was defined to occur at peak foot plantarflexion occurred on the ipsilateral side (Figure 2D).

The setting of each event was verified in the software by visual inspection of the joint angle and velocity curves and skeleton animations and was manually adjusted if the automatic event identification was deemed incorrect (Figure 2). This routine is necessary to avoid erroneous events due to noise or artefacts and the same routine is commonly used in gait laboratories [24]. The first and last event represented motion initiation and motion ending and was excluded from each trial. Kinematic data was then time-normalized over each gait cycle and variables were computed for each gait cycle for the pelvis and thorax segments and for the shoulder, hip, knee, and ankle joints. The variables were selected from kinematic and temporal variables earlier suggested for use in clinical gait analysis [26] and of interest for post-stroke analyses [7,8,10,11,12]. These variables were the peak flexion angle, peak extension angle, the range of motion (i.e., peak flexion to peak extension, RoM), and the stride frequency (number of gait cycles per second). An average value from all gait cycles was computed for each variable and each test session and used for the statistical analyses.

### 2.5. Statistics

IBM SPSS^®^ Statistics 27 and Microsoft Excel 365 were used for statistical analysis. Kolmogorov Smirnov was used to test the normal distribution of the included variables. As only 10 of 52 variables failed the normality test (*p* = 0.00–0.04), means and standard deviations were chosen for graphical presentation of the time-normalized data.

For consistency, variables from the dominant (i.e., the right) side were further analyzed for test-retest reliability. An exception was made for variables from the shoulder angle during stair walking, where the left side was used instead because all participants used the right hand to grab the handrail.

Bland–Altman plots were constructed to explore the agreement of the test-retest measurements. The test-retest reliability for the two test sessions was analyzed with the intraclass correlation coefficient, ICC (3, k, absolute agreement) [27]. The ICC value was computed from the averaged kinematic and temporal variables from each of the two sessions. The 95% confidence interval for ICC was 0.50–0.75 moderate reliability, 0.75–0.90 good reliability, and >0.90 excellent reliability [27].

## 3. Results

**Figure 3:**
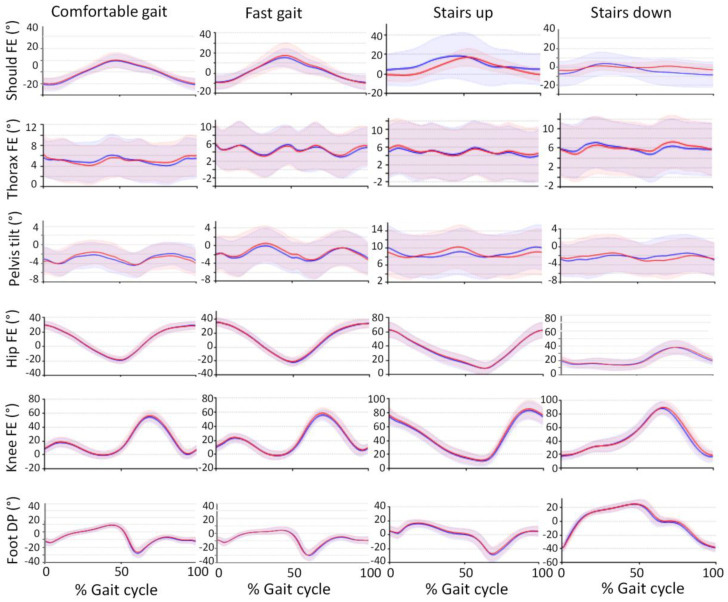
Group average joint angular curves, constructed from each participant’s mean curve during comfortable gait, fast gait, and stair walking. Data were time-normalized over each gait cycle (i.e., the time sequence between two successive events). The figure shows sagittal plane movement, i.e., shoulder, thorax, hip, and knee flexion-extension, pelvis anterior-posterior tilt, and foot dorsiflexion-plantarflexion. Blue curves represent angular movement during right-sided (dominant) gait cycles and red curves represent angular movement during left-sided (non-dominant) gait cycles. The right leg and arm are displayed for right-sided gait cycles, and vice versa. Shaded areas represent the group standard deviation.

Eight trials were excluded from the analyses because of sensor drift. In all these cases, the participant failed to stand still in the standardized position during the initial part of the trial, which led to the initial sensor calibration failing, giving a typical drift of several degrees per second. Seven gait cycles were excluded since the participant was disturbed by outer circumstances, such as people passing in the corridor. The total number of gait cycles available for further analyses were 873 (comfortable gait), 719 (fast gait), 479 (stair gait ascending), and 504 (stair gait, descending). The average joint and segment angle curves are displayed in Figure 3. The figure displays a high similarity between the dominant and non-dominant side’s gait cycles, with an exception for the shoulder joint motion during stair walking, when a side difference appear as the right hand was held on the handrail. It also shows similar gait patterns for the comfortable and fast gait but with a decreased shoulder range of motion and slightly increased hip and knee range of motion at the faster speed. When ascending the stairs, a different but repetitive walking pattern compared to gait was observed, with less hip and knee extension and greater knee flexion (Figure 2).

**Figure 4:**
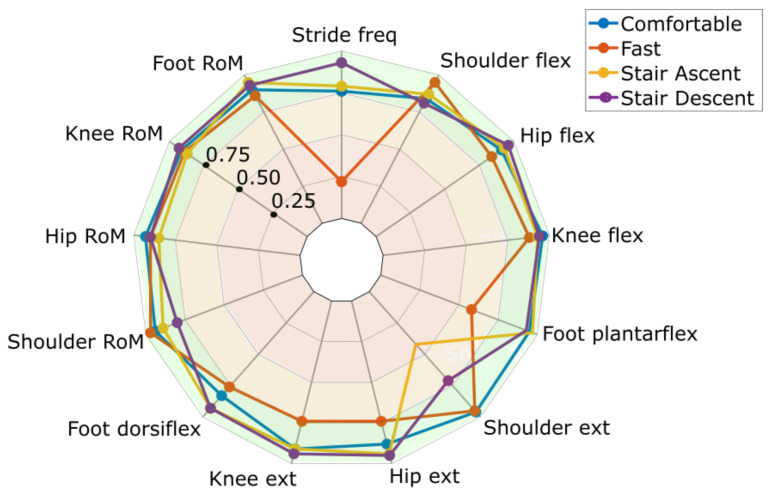
Intraclass correlation coefficients (ICCs) for test-retest measurements of peak angles, range of motion (RoM), and stride frequency during comfortable gait (blue), fast gait (red), stair ascending (yellow), and stair descending (purple). ICC < 0.5 (red shaded area) represents poor reliability, ICC from 0.5 to 0.7 represents moderate reliability, and ICC < 0.75 (green shaded area) represents good to excellent test-retest variability.

**Figure 5:**
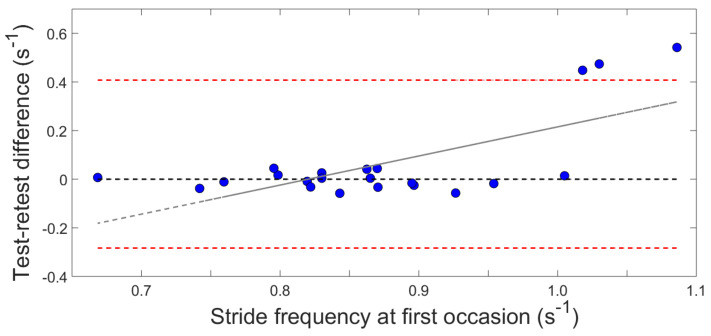
Bland–Altman plot of the stride frequency during gait at a fast speed. The differences in the stride frequency between the test-retest measurements in relation to the stride frequency during the first test session are shown for all 22 participants.

The test-retest repeatability was analyzed for the right-sided gait cycles. The ICC values are displayed in Figure 4. The test-retest reliability when walking at a comfortable speed was good to excellent for all variables (ICC 0.83–0.96). The same result was found for the fast gait variables (ICC 0.76–0.97) except for hip and knee extension (ICC 0.74), foot plantarflexion (ICC 0.58), and stride frequency (ICC 0.22). The Bland–Altman plot showed that mainly 3 out of the 22 participants contributed to this high between-session variability in stride frequency (Figure 5). Shoulder peak extension had poor to moderate reliability during stair walking (ascending: ICC 0.42, descending: ICC 0.71) while the other variables had good to excellent reliability (ascending: ICC 0.85–0.97, descending: ICC 0.80–0.96).

## 4. Discussion

### 4.1. Test-Retest Reliability

In this study, a test battery for clinical evaluation of gait speed during comfortable gait, fast gait, and stair walking was extended to involve kinematic and temporal measures from the lower body and arms, based on data from a wearable inertial sensor system. This study showed high test-retest reliability for all ranges of motion and peak angles in the sagittal plane during comfortable gait. This is in agreement with Orlowski et al., who used a simpler model (inertial sensors on the shank, pelvis, and thorax) to show excellent test-retest reliability for pelvis and spine angular movement during gait [28]. When walking at a fast speed, moderate reliability was found for hip and knee extension and foot plantarflexion, with poor reliability found for stride frequency. This is probably because some individuals had a different stride frequency in the first compared to the second session, since gait speed affects joint kinematics [29]. If fast gait is to be evaluated, one choice could be to standardize the gait speed with a metronome, but with the risk of affecting the individual’s own appreciation of fast gait. Another alternative could be to change the instructions given to the participant to be more similar to the 25FWT [4].

We showed in an earlier study that the inertial sensor-based system was reliable for assessing the range of motion in the arm and upper body during arm function tests [30]. In the current study, the reliability in shoulder extension was excellent when walking at a comfortable and high speed but was poor when ascending stairs and moderate when descending since we allowed the participants to use the handrail to simulate more natural stair walking. This is how the task must be designed for patients with neurological deficits to avoid falls. The arm swing during level gait is of interest when studying stroke patients [11], so based on these results, we recommend the study of shoulder kinematics during comfortable and fast gait but not during stair walking.

Range of motion showed the best overall reliability. One explanation could be the assumption that all joints are aligned at zero angles in the standardized position. If the participant stood with slightly flexed legs during the standardized position sequence, this may have resulted in a systematic difference in the calculation of the peak angles, without affecting the calculated range of motion in each stride. It is necessary to analyze patient groups with neurological disorders to be able to decide whether such systematic differences in peak angles are acceptable in relation to the magnitude of clinical differences, e.g., prior to and after rehabilitation.

In this study, we chose to include one temporal measure, the stride frequency, which was good to excellent for comfortable gait and stair walking. This confirms earlier studies showing good test-retest reliability for stride time based on inertial sensors [14,31,32].

### 4.2. Methodological Aspects

The sensor placements were chosen to be repeatable and to avoid soft tissue artefacts as far as possible. The sensor placements on the thorax and arms were selected based on an earlier study that analyzed optimized sensor positions [18]. The suggested positions for the pelvis and thigh segments were similar to a study by Niswander et al. [33]. This study suggested that the foot sensor could be placed either on the dorsal foot (as in our study) or on the heel, and that the shank sensor should be placed in a lateral position [33]. Since the persons in the current study walked barefoot, it was judged that the dorsal foot position was more appropriate. We also judged that the frontal position on the shank was better than the lateral position to avoid artefacts from the calf muscle. A lateral placement is common when analyzing shank movement in optical gait laboratories to avoid marker data loss during shank flexion, but this is not an issue when using inertial sensors.

The sensor-to-segment alignment was based on a single step calibration, where the participant stood still in a standardized position. The standardized position used in the calibration procedure has limitations. For example, the knee flexion angle in this neutral position may vary by several degrees for different individuals, and further, patients with stroke may have difficulties standing in a straight position due to paresis and spasticity. This suggests that range of motion may be more reliable than absolute peak angular positions for the current methodological setup. Other calibration procedures could be implemented to improve the sensor-to-segment alignment, such as a functional calibration where the participant performs a set of standardized leg and body movements to align the sensors’ coordinate systems [34]. However, the drawbacks are that this procedure is more time-consuming, and the standardized movements may be difficult for a patient to perform, which in turn introduces alignment errors. Magnetometers further improve the alignment [35] but are not suitable in the hospital environment due to the high risk of magnetic disturbances.

The sensors’ three-dimensional orientation was calculated using the Madwick fusion algorithm based on a quaternion representation [25]. This algorithm was used in an earlier study for kinematic calculations and validated against an optical reference system [30]. It was also compared with nine other algorithms by Caruso et al. [20], showing that the filter parameter settings seem more important for the performance than the choice of the algorithm. The filter parameters in the current study were tested for gait and other lower body movements in an earlier study in comparison with an optical system [23].

The only event included in the analysis was the initial contact (heel-down during level walking and toe-down during stair walking). All kinematic parameters were calculated within each stride based on this event. Other phases, such as stance phase, swing phase, and support phase, were not analyzed. The main reason was that all events were visually verified, due to the risk of erroneous event setting for atypical gait patterns. Hence, this is the most time-consuming part of the analysis procedure. Orlowski et al. showed high test-retest reliability for stride time but low test-retest reliability for the stance and swing phases [28], which also indicates that it may be more demanding to correctly define the phases within each stride, at least based on inertial sensor data. Still, this single initial contact event allows the calculation of several temporal aspects with importance for neurological disorders, such as stride frequency, stride variability, temporal side asymmetries [10], and temporal adaptions during fast gait [12].

The initial contact can be defined in different ways, depending on the type of measurement method and number of measurement units. Event setting based on shank and foot velocity is more accurate compared to algorithms based on lower trunk movement according to a review [19]. Zahradka et al. analyzed three different algorithms to detect the initial contact in comparison to a gold standard method (force plate): a foot velocity algorithm, a shank velocity algorithm, and a kinematic algorithm based on pelvis, shank, and foot markers [32]. This study showed that any of the algorithms could be used reliably, but the shank velocity algorithm was suggested as an easy-to-implement method when using inertial sensor systems. Regardless of the choice of the event-setting method, we still recommended a semi-automatic procedure, if the method is used in a clinical context, since there is always a risk that the automatic algorithms will fail when analyzing patients with avoiding gait patterns.

### 4.3. Clinical Implications

Instrumented three-dimensional (3D) gait analysis based on optical camera systems (the gold standard method) can identify gait deviations in a way that is not possible from clinical observation alone [21,36], but the method is rather time consuming, expensive, and requires specialized personnel and is therefore unavailable for many patient groups. If implementing a gait analysis method in the clinical routine, the method needs to be time-efficient and reliable when performed by the clinician, without referral to a specific laboratory.

The test battery in the current study is commonly used for evaluation of gait function in neurological patients and was extended to include a wearable inertial sensor system for a more thorough gait analysis. The kinematic and temporal outcome measures can be used to evaluate, e.g., gait asymmetry and reduced range of motion, which are important aspects of walking ability post-stroke [7,8,9,10,11,12]. However, the current study was based on a control group with normal walking ability, and the results needs to be verified in patients with neurological disorders, such as stroke.

The implementation was rather straight-forward, and physiotherapists in the current study could perform the measurements and analyses after a few training sessions. Even so, the time consumption from measurement to a ready analysis was still about 40 min in the actual configuration. To avoid unnecessary patient fatigue, it could be beneficial to simplify the measurement and analyses procedures even further, for example, by excluding arm sensors and using the lower body model only, and/or selecting one of the three gait tests, depending on which outcome measures are most important in relation to the specific patient’s gait severity.

## 5. Conclusions

The inclusion of a wearable inertial sensor-based system in a clinical gait test battery provides kinematic and temporal outcome measures with good to excellent test-retest reliability, with a few exceptions, such as stride frequency during fast speed and shoulder kinematics during stair gait. The method has the potential to improve the clinical evaluation of gait function, but this needs to be confirmed in patients with neurological disorders.

## Data Availability

Data are available, please contact the authors by e-mail.
